# Auer Rods in Chronic Myelomonocytic Leukemia Can Change the Diagnosis

**DOI:** 10.4274/tjh.2015.0148

**Published:** 2015-08-01

**Authors:** Smeeta Gajendra, Ranjit Kumar Sahoo

**Affiliations:** 1 Medanta-The Medicity, Department of Pathology and Laboratory Medicine, Haryana, India; 2 All India Institute of Medical Sciences, Department of Medical Oncology, New Delhi, India

**Keywords:** Auer rod, Chronic myelomonocytic leukemia, Hematopoietic stem cell transplantation

## TO THE EDITOR

**Figure 1:**
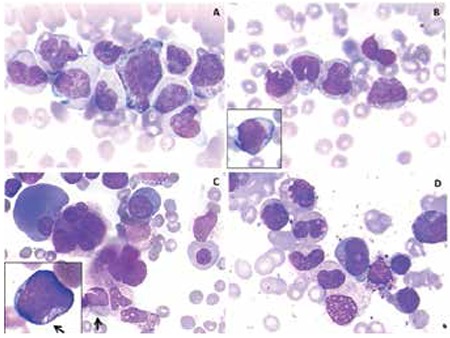
A, B) Peripheral blood smear showing presence of abnormal monocytes along with myelocytes and blast (B- inset). C, D) Bone marrow aspirate showing dyspoiesis in all three lineages (C) with an occasional blast showing an Auer rod (C, arrows) with increased monocytic cells (D) (Jenner and Giemsa stain, 1000x).

Chronic myelomonocytic leukemia (CMML) is a clonal hematopoietic stem cell disorder with overlapping morphological features of myelodysplastic and myeloproliferative disease and a potential risk of transformation to acute myeloid leukemia. Presence of Auer rods in CMML is a rare finding and the presence of an occasional Auer rod gives the diagnosis of CMML-2 in spite of the presence of <5% blasts in peripheral blood/bone marrow [1,2]. A 39-year-old female, diagnosed outside our facility with Crohn’s disease, presented with severe anemia with weakness and fatigue for 1 month. The patient had been treated previously with prednisolone at 1 mg/kg/day. Diarrhea was resolved after 2 weeks of therapy. Steroid dose was reduced and stopped after 4 months. There were no bowel symptoms and the colonoscopy done at our institution was normal. Hemoglobin was 65 g/L, total leukocyte count was 16.4x109/L, and platelet count was 192x109/L. Peripheral blood smear showed 4% blasts and promonocytes, 3% myelocytes and metamyelocytes, 26% monocytes (including abnormal forms) (Figures 1A and 1B), and 2 nucleated red blood cells/100 white blood cells. Bone marrow aspirate was hypercellular with dyspoiesis in all 3 lineages with increased monocytic cells (Figures 1C and 1D). Erythroid series showed predominantly megaloblastoid erythropoiesis with nuclear budding, multinuclearity, and cytoplasmic vacuolation. Granulocytic series showed myeloid hyperplasia with 15%-20% monocytes, 2% basophils, and 4% blasts, with an occasional blast showing an Auer rod (Figure 1C, arrows). Micromegakaryocytes and megakaryocytes with abnormal lobation and multinucleation were seen. Bone marrow biopsy was hypercellular (100%) with grade 1 reticulin fibrosis. Megakaryocytes were increased in number and showed hypolobation and multinucleation. Conventional cytogenetics showed a normal female karyotype. There was no Philadelphia chromosome or BCR/ABL fusion gene. Overall features were compatible with a diagnosis of CMML type 2. She was started on 3+7 induction chemotherapy using daunorubicin and ara-C. Bone marrow aspirate done on day 28 confirmed morphological complete remission. She underwent HLA-identical allogeneic hematopoietic stem cell transplantation from her elder brother and currently (2 years posttransplant) continues to be disease-free. Auer rods are a hallmark of acute myeloid leukemia but are occasionally seen in myelodysplastic syndrome (refractory anemia with excess blasts type 2) or CMML cases, and rarely in patients with fewer than 5% blasts [3,4]. According to the World Health Organization 2008 diagnostic criteria, the presence of Auer rods fulfills the criteria for CMML-2 irrespective of the blast count [5]. Thus, in CMML, a thorough search for Auer rods should be done for a correct diagnosis as the treatment given for CMML-2 is different from that for CMML-1 and the risk of transformation to acute leukemia is greater. We also want to emphasize that the presence of Auer rods with fewer than 5% blasts is a rare phenomenon that seems to be clinically, morphologically, and cytogenetically heterogeneous, and it could be a valuable finding for early treatment options in patients with CMML-2 if there is a HLA-identical donor.

**Conflict of Interest Statement**

The author of this paper has no conflict of interest, including specific financial interests, relationships, and/or affiliations relevant to the subject matter or materials included in this manuscript.
